# Acute flaccid myelitis a review of the literature

**DOI:** 10.3389/fneur.2022.1034607

**Published:** 2022-12-20

**Authors:** Darina Dinov, Jeffrey R. Donowitz

**Affiliations:** ^1^Department of Neurology, Children's Hospital of Richmond at Virginia Commonwealth University, Richmond, VA, United States; ^2^Department of Pediatrics, Children's Hospital of Richmond at Virginia Commonwealth University, Richmond, VA, United States

**Keywords:** child neurology, acute flaccid myelitis, acute flaccid paralysis, enterovirus, muscle weakness

## Abstract

Acute flaccid myelitis (AFM) is a rare neurological disorder that first rose to national attention in 2014. This neurological disorder has a biennial presentation with every other even year being a peak year. Most patients present in childhood 5 days after a prodromal infection. Patients usually present with muscle weakness and hypo or areflexia in the summer or fall months. Clinical outcomes are variable however most patients do not improve. Currently there are no definitive prognostic factors or etiologies found. However, it is thought that enterovirus-D68 (EV-D68) could be a potential component in the pathobiology of AFM. Treatment options are limited with variable options and no consensus. Supportive therapy has been shown to be the most effective thus far. With our review of the literature, we highlight the recent growing evidence of a possible relationship between EV-D68 and AFM. Additionally, we identify the knowledge gaps in AFM with treatment and prognostic factors.

## Introduction

Acute flaccid myelitis (AFM) first rose to the attention of the generalized medical community in the spring and summer of 2014, when there were clusters of children with flaccid paralysis predominantly in California, Utah, and Colorado. AFM is a neurological disease which causes muscle weakness and hyporeflexia in children (1). This disease has a biennial pattern with case spikes every even year, defined as peak years (2). It is postulated that this biennial presentation is due to viral susceptibility and a population size large enough that does not have enough antibodies to fight the virus. Nevertheless, there is no definitive data on what causes this biennial presentation. Most cases present with a prodromal illness followed by weakness predominantly in the upper extremities (3–6). There are no proven effective therapies for AFM and the cornerstone of treatment remains in supportive care with physical, occupational, and other therapies. Clinical outcomes are variable and age, presentation, ethnicity, geographical location, laboratory studies, and imaging study results have not been associated with outcome (5–9). Currently there are no identified indicators of AFM prognosis.

## Clinical presentation

The mean age of presentation for AFM is 5.8 years of age, with most cases in children ranging from 2 to 11.5 years with a male predominance (3, 6, 9–11). Patients generally presented 5 days after symptom onset (ranging from 2 to 7 days) in the late summer and fall in a biennial pattern in even years (3, 5, 8, 9). However, with the COVID-19 pandemic it is unclear if this pattern will continue.

When comparing peak verses non-peak years (defined as odd years), it is noted that mean age was significantly lower in peak years, 5.2 years verses 8.3 years in non-peak years (12). This could help support the claim that the virus needs a new cohort without pre-formed antibodies to result in AFM. Therefore, in non-peak years young children likely have herd immunity due to previous infections so will not be affected, while older children did not obtain herd immunity. The ethnicity most affected is Caucasian (3, 13) with Asian being second most common (9–11). Most patients did not have any significant medical history; however, in those that did asthma was the most common, ranging from 10 to 25% in most studies (8–10, 14). Further, etiology evaluation in relation to presentation will be discussed in the etiology section of this manuscript.

Most patients with AFM initially present with respiratory symptoms including rhinorrhea, cough, pharyngitis, and fever (2, 5, 8, 9). In numerous studies or reviews >80% of patients initially present with viral respiratory symptoms at the time of diagnosis (6, 7, 11, 15). Progression to respiratory failure is common in these cases and there are reports of patients requiring a tracheostomy for support (3, 6, 8, 9). In comparison 30–64% of patients have gastrointestinal symptoms, usually vomiting and diarrhea (6, 13, 16). While over half of patients had a fever (5, 13). Other significant findings on presentation include neck stiffness noted in 0–60% of patients (9, 17, 18), bladder or bowel dysfunction noted in 5–40% of patients (18), altered mental status found in < 20% of patients (3, 13), and seizures found in < 10% of patients (3, 13). All these prodromal factors would support the thought that AFM pathobiology is at least partially attributed to a pathogen.

An interesting point to note is that respiratory symptoms were more frequently found in peak years while GI symptoms were noted almost equally in peak and non-peak years (12). Febrile episodes differ in peak years verses non-peak years with 72% of patients in a peak year being febrile verses 52% in non-peak years (12). The cohort these findings are based on is a US nationwide cohort comparing pediatric patients from 2015 to 2018. It is unclear why patients in peak years would generally present with more infectious symptoms. One explanation could be that those in peak years have less antibodies to enterovirus-D68 (Ev-D68) or other viruses. This would support the biennial pattern hypothesis in AFM.

In reports or manuscripts published on patients in Argentina, China, and Japan authors did not find a significant difference in their patient population compared to the United States population (19–22). Most patients had febrile episodes and respiratory infections like those in the United States (19–22). This is important to note because it further supports a possible viral infection, like EV-D68, as a component in the pathobiology of AFM.

Physical examination was significant for asymmetric limb weakness more pronounced in the upper extremities that developed over several days (5, 9). In the study by the CDC comparing patients from 2015 until 2018 in peak verses non-peak years authors found that that lower extremity was present more frequently in non-peak years (12). Overall, weakness was more predominant in the proximal muscle groups (3, 5, 9). Frequently, up to 55% of cases, limb weakness was present in 1 or 2 limbs (3, 13). This differ somewhat in non-peak years where four limb paralysis is noted more frequently than peak years (12).

AFM patients present with cranial nerve involvement less than weakness in other regions (5, 7, 19, 23). Cranial involvement has been noted to occur up to 26% of cases (7, 12, 19). If involved the most common cranial nerves involved were the facial, followed by the abducens, and the oculomotor (3, 24, 25).

Deep tendon reflexes (DTRs) were absent or decreased in most patients (5, 9, 24). Hyporeflexia or areflexia were reported in up to 80% of patients (9, 17). Sensory involvement was less common, occurring in < 10% of patients but ranging from 0 to 45% across various studies (6, 17, 18). This emphasizes the importance of a comprehensive physical examination, particularly a neurological examination, for the provider to consider AFM on the differential.

## Etiology

Currently, there is no clear causative factor for AFM. The presentation of AFM is similar to poliovirus and other acute flaccid paralysis cases leading to the thought that this is secondary to an infectious etiology as those are. The pathobiology of AFM through an infectious etiology can be explained through molecular mimicry. This process occurs when a pathogen activates a T cell response and self-antigens erroneously develop through the appropriate immune response (26). These self-antigens can create an autoimmune process once they are activated by the same pathogen at a later date. They do this by activating along with the T cells so both an adaptive and autoimmune attack occurs (26).

Although, enteroviruses (EV) are not always identified in cases of AFM they are the most implicated virus with a positivity rate of 20–96% in patients with a viral test (3, 5, 6, 9, 13, 27, 28). Enteroviruses peak in the late summer to fall just as AFM cases do (29, 30). This peak amongst enterovirus cases and AFM cases would further support the claim that EV is involved in the pathobiology of AFM. When applying the Bradford Hill criteria, which can establish a causal relationship between a cause and effect, EV-D68 has been shown to have a causal relationship with AFM (3, 5, 28, 31).

Further support for this is found in tissues that contain EV-D68 or are exposed to it directly. In mouse modules when subjects are injected with the virus, or they have an intranasal exposure the virus is found in the motor units of the spinal cord and neuromuscular junction (32). Ultimately, these mice are found to develop acute myositis and paralysis in their limbs (32). Thus, far there has only been one case of EV-D68 being found in the spinal tissue of a patient diagnosed with AFM. This was in a 5-year-old patient diagnosed in 2008 where authors found EV-D68 RNA and protein in post-mortem autopsy in his anterior horn motor units (33). This further supports the hypothesis that EV-D68 plays a role in the pathobiology of AFM.

In a study from 2015 to 2018 authors compared peak to non-peak years for the rate of rhinovirus/enterovirus positivity. The authors of this study found a higher positive rhinovirus/enterovirus case number in peak years, 38%, verses in non-peak years, 19% (12). This supports the thought that the biennial presentation is due to viral circulation in peak years causing antibodies that take effect more in non-peak years ultimately causing less infections. An alternative thought is that an alternative strain of EV-D68 develops with a new viral capsid which is more pathogenic since the number of cases increase with every biennial presentation. This needs to be studied further to truly evaluate.

In non-peak years the virus that is most found is EV-D71 (12). Compared to EV-D68, EV-D71 was found to be positive 20% in peak years verses 13% in non-peak years (12, 18). This is a virus that is in the same family of picornaviruses as EV-D68. It has similar functions as EV-D68 in viral replication, autophagy, inhibition of the inflammatory pathway except it contains a few more proteins that help with autophagy (34).

Other pathogens which have been associated with AFM include Adenovirus, Epstein-Barr virus, West Nile virus, Human Herpesvirus 6, and Mycoplasma (2, 5, 9, 13, 35). In a study evaluating cases form 2012–2015 authors found that EV-D68 was positive in 22% of the cases while other viruses including EV-71, West Nile virus, and Japanese encephalitis virus were positive 18% of the time (5). Comparatively, a CDC study in 2018 evaluating patients found that echovirus 11 caused one case of AFM while coxsackievirus caused three and paraechovirus caused four (2).

The majority of those tested in the above studies were done by nasopharyngeal swabs. In a study evaluating 123 patients' multiple pathogens other than EV-D68 were found: 6 patients had a CSF positive for adenovirus, Epstein-Barr virus, HHV-6, or mycoplasma (13). These variable pathogen detections emphasize the importance of a complete workup for patients as well as the variability in pathogens with the ability to trigger AFM.

## Evaluation

The Center for Disease Control (CDC) definition to help diagnose cases of AFM requires acute onset flaccid limb weakness and an MRI with a spinal cord lesion in the gray matter or spanning multiple segments (36). Serum studies were significant for mild-to-moderate leukocytosis and elevated inflammatory markers (9, 37). Serum positivity rates for viral studies were < 5% (13). The majority of viral positive results were from nasal swabs (3, 5, 6, 9, 37). Viral serology studies were discussed further in the etiology section above.

Cerebrospinal fluid (CSF) studies showed pleocytosis, with a mean of 44 white cells/μL, with lymphocytic predominance and an elevated protein count (3, 5). Lymphocytic predominance is consistent with a viral etiology. However, most cases did not present with a positive viral test in the CSF (3, 5, 18, 38). The ones that did, < 5% of cases, were positive for EV-D68 (3, 5, 9).

Stool studies for viral identification were commonly conducted, but negative most of the time (3, 5, 9, 13). In the United States, < 10% of stool studies were positive compared to Europe, Argentina, and China which had a positive rate of 35% (5, 19–22). Most international evaluations were based on 2016–2018 data compared to the 2014–2016 United States data. It is unclear why this difference in stool positivity rates occurred; was it due to year of analysis or countries of analysis. Further diagnostics of this are needed to elucidate this relationship more.

Imaging findings across the studies were consistent with many patients, about 90%, having T2 gray matter non-enhancing multi-level spinal cord lesions worst in the anterior horn (5, 9, 18, 27). The most affected region is the cervical spine, affecting up to 87% of patients, followed by the thoracic spine which is present in about 80% of patients (3). Nerve root lesions in the MRI occur sporadically ranging from 0 to 72% of cases (3, 9). The most common location of brain lesions was the pons or medulla (2).

Most patients did not get an electromyography (EMG) or nerve conduction studies (NCS) since characteristic findings do not emerge until later in the course. The patients that did get NCS showed a reduction in the compound mean action potentials and reduced amplitude with preserved sensory nerve action potentials (22, 29, 39–41). EMG showed a reduced recruitment of motor unit action potentials consistent with anterior horn damage (22, 29, 39–41).

## Management

There is currently no evidence based standard guidelines or expert consensus documents for the treatment of AFM. Many patients receive intravenous immunoglobulin (IVIG), steroids, plasmapheresis, or fluoxetine yet these have not shown a benefit (3, 5, 6). Fluoxetine was originally thought to work by reducing the viral load through the reduction of EV-D68 replication; however, studies have shown that it is not effective (17, 27). Antivirals overall have shown little benefit in AFM and are not routinely used (17, 27, 42).

Most studies to evaluate the treatment potential of steroids have been conducted in mouse models. In one study done on mice when injected with steroids the viral load would increase (5, 43, 44). This would indicate that steroids are actually worse for treatment, however this has not been demonstrated in human EV-D68 AFM cases (5, 43, 44). Interestingly, poorer outcomes have been documented in AFM patients infected with EV-A71 who received steroids (44). Due to this the World Health Organization and CDC recommend avoiding steroid use for patients who have AFM with an EV-A71 infection (24, 25, 37).

IVIG is used frequently in AFM with variable results (45). It presumably works by reducing the viral load and by affecting cytokine production, primarily blunting the immune response. Presently, there have been no clinical studies conducted on the efficacy of IVIG for AFM. Case reports suggest IVIG is beneficial for some patients, with some IVIG samples even containing antibodies for EV-D68 (46). However, effectiveness depends on the amount of anti-EV neutralizing antibodies for the specific infecting serotype (46).

Plasmapheresis has not been extensively evaluated. Since it works by removing the autoantibodies decreasing the viral immune response it is thought to be beneficial by removing anti-enterovirus antibodies (5, 6). The therapies discussed above are thought to be potentially effective if there is a viral or autoimmune component in the pathobiology of AFM.

Nerve transfers either end-to-end transfers or side-to-end nerve transfers are another potential option with limited data (47, 48). One study showed patients improved from a manual muscle testing score of 0–3 (48). In one case study four out of five children were able to ambulate again with an orthotic after nerve transfer (48). Patients that received nerve transfers for shoulder external rotation and elbow flexion had excellent outcomes in >87% of cases compared shoulder abduction nerve transfers (47). However, with nerve transfers it is imperative that this occurs within 18–24 months of symptom onset (48).

The most effective treatment options presently are physical and occupational therapies. These therapies have not shown significant improvements in terms of muscle strength or regaining the ability to walk; however, they have shown plateauing and even minimal gain in muscle strength and functional gains (49–51). In order to truly find an effective treatment head-to-head comparisons of treatment options should be done.

## Prognosis

The prognosis for AFM is poor as there is no effective treatment which prevents muscle weakness. Recent studies have shown that 5–39% of patients regain some motor function (2, 5, 6, 25, 29). Yet, the bulk of patients the authors have seen or read about have had significant motor deficits. Studies have found no correlation between detection of EV-D68, EV-A71, laboratory, or imaging findings with prognosis (3, 5, 6). Some reports note the severity of deficits on EMG or NCS correlate with disease course or outcome however the cohorts evaluated are too small to make generalized claims (39–41, 52).

## Conclusion

AFM is a relatively new diagnosis, first noticed in 2014, although acute flaccid paralysis (AFP) has been documented in pediatric patients for a long time. The literature currently supports a seasonal, biennial pattern for AFM cases. There is supportive evidence to indicate that AFM cases continue to rise in peak, even, years. No definitive instigating factors have been noted. Based on the Bradford criteria, a biopsy in a previous AFM patient showing EV-D68 in the anterior horn cells, and temporal associations of EV with AFM it is likely that EV-D68 has a role in the pathobiology of AFM although it does not appear to be the only pathogen capable of causing this disease. It is likely the pathobiology is multifactorial given the various other viral and bacterial etiologies which have been detected in AFM cases particularly in peak verses non-peak years.

## Future directions

There are several knowledge gaps for AFM that create barriers to treating these patients. As discussed previously- the etiology and pathobiology remain unclear, there are no known definitive prognostic indicators for AFM, and there is no consensus on treatment. Current summarized information does indicate a possible relationship between EV-D68 and AFM, with AFM potentially caused by EV-D68. This needs to be analyzed further to truly establish this correlation. There are two ongoing biorepositories being conducted by the National Institutes of Health (NIH) and the Centers for Disease Control and Prevention (CDC) which may answer some of these questions (53). The relative rarity of AFM is a barrier to research and will necessitate coordinated multicenter studies such as those conducted by the NIH and CDC. Future work should focus on both the etiology and treatment as well as identification of prognostic indicators to guide care.

## Author contributions

Material preparation, data collection, and analysis were performed by DD and JD. The first draft of the manuscript was written by DD. All authors commented on previous versions of the manuscript, contributed to the manuscript conception and design, read, and approved the final manuscript.
